# Knowledge and attitudes regarding medical research studies among patients with breast cancer and gynecological diseases

**DOI:** 10.1186/s12885-015-1584-3

**Published:** 2015-08-14

**Authors:** Michael P. Lux, Thomas Hildebrandt, Sandra-Maria Knetzger, Michael G. Schrauder, Sebastian M. Jud, Alexander Hein, Claudia Rauh, Peter A. Fasching, Matthias W. Beckmann, Falk C. Thiel

**Affiliations:** Department of Gynecology and Obstetrics, Erlangen University Hospital, Comprehensive Cancer Center Erlangen-EMN, Friedrich Alexander University, Universitätsstrasse 21-23, 91054 Erlangen, Germany

**Keywords:** Clinical study, Clinical trials, Questionnaire, Study participation, Patients’ knowledge, Patient information, Recruitment

## Abstract

**Background:**

Medical research studies are becoming increasingly important for optimizing the prevention, diagnosis and treatment of illnesses. Participation in research studies can have many benefits for patients. In randomized and controlled clinical studies, they can receive the best possible medical care currently available. However, only a small proportion of patients nowadays are treated within the framework of medical research. The primary endpoint of this study was to discover what level of knowledge patients have about clinical studies and how they currently perceive them, in order to identify ways of optimizing the information provided about studies from the patients’ point of view.

**Methods:**

The study included 2546 patients (breast cancer 21.6 %, gynecological cancer 8.3 %, obstetrics 32.7 %, endometriosis 7.8 %, fertility treatment 3.2 %, other benign gynecological illnesses 19.2 %, no information for 7.2 %) in the outpatient clinic (45.2 %) and in the in-patient sector (54.8 %) at the Department of Gynecology at Erlangen University Hospital and associated centers. In the single-center study, conducted between January 2011 and January 2012, the patients were asked about their level of knowledge regarding the background to medical research studies and the ways in which they are carried out and used. The patients were also asked how they perceived medical studies and how they thought study conditions might be optimized. The three-page questionnaire was included in the feedback sheet received by patients as part of the hospital’s quality management system.

**Results:**

As a whole, the group only had moderate knowledge about clinical studies. A majority of the respondents considered that studies were valuable (91.6 %), but only a few were also willing to take part in them (58.4 %). Knowledge and willingness to participate strongly depended on age (*P* < 0.001), educational level (*P* < 0.001) and patient group (*P* < 0.001). Most patients would prefer to decide about participating in studies through a discussion with their outpatient physicians.

**Conclusions:**

The information that patients have about clinical studies affects whether they participate in them. It is therefore extremely important for patients to be well informed, for their anxieties about participation to be relieved, and for the benefits of participation to be explained to them.

## Background

Medical research studies are today the gold standard for the development of new types of medical procedure, treatments, drugs, etc. They help to transfer the latest scientific discoveries to clinical practice. They therefore have great potential benefits for patients who participate in them, and thanks to strict standards and regulations they have very high safety requirements. Treatment in the context of a clinical study is claimed to be equivalent to or better than the standard treatment and implies increasingly new therapeutic options, particularly in the field of oncology [1]. In addition, the results of further studies suggest that patients treated in research studies have an improved quality of life [2]. Care for participants in a clinical study is much more intensive, and more information is provided to the patient about the causes and management of disease; this leads to an additional positive psychological effect [3]. Despite this, however, participation rates in clinical studies are extremely low [4].

To increase the numbers of patients participating in research studies, both an optimal study design and also adequate provision of information are extremely important. It is therefore of decisive importance to know from the patients’ point of view what level of knowledge they have about clinical studies and how they currently perceive them, and to know about ways they think the information provided could be optimized.

The present study investigated the general level of knowledge on the topic of “clinical studies” in a group of patients being treated at a university gynecology department. In addition, the way in which they perceived the design and conduct of studies and their attitudes to them were also noted.

## Methods

Between January 2011 and January 2012, outpatients and in-patients at the Department of Gynecology at Erlangen University Hospital were asked to complete a questionnaire to assess the level of knowledge they had about clinical studies and how they perceived them, as the primary endpoint. The secondary endpoint was the analysis of differences between the subgroups with different diseases.

The questionnaire included 25 questions and was linked to the survey about patient satisfaction, given to all patients in the department as part of quality management. The questionnaire was filled out at the end of the outpatient visit or in-patient stay, to avoid stress for patients who might be waiting for a diagnostic or therapeutic procedure.

The first section of the questionnaire covered their knowledge relative to nine statements about clinical studies; the patients had to assess the truth of these statements. The second section consisted of 13 closed questions, each with the optional answers “yes/don’t know/no.” The questionnaire also included three open questions on the topic of the value of clinical studies and factors potentially influencing participation in them. In addition to these data, the condition being treated, the patient’s highest educational level, and age and time since first diagnosis were also recorded. After the questionnaire had been distributed for 4 weeks initially, the pattern with which patients completed the questionnaire and its comprehensibility were checked. During this validation phase, no evidence was found that the questionnaire was difficult to understand.

The study was approved by the Ethics Committee of the University of Erlangen.

In addition to direct analysis of the responses to the nine individual statements, four of the statements were prospectively selected as being clearly correctly answerable, and the numbers of correct answers from each patient were added up to a create a score (0–4). The score was used as a separate indicator of the patients’ knowledge about clinical studies, comparable to the knowledge scale developed by Ellis and colleagues [5]: “Do you know what the word ‘randomization’ means?” (answer: “yes”); “Clinical studies make it possible to find out whether the standard treatment or a new method is more effective” (answer: “yes”); “Research studies cannot be used in the treatment of severe diseases” (answer: “no”); and “After you agree to take part in a study, withdrawing from it later on is no longer possible” (answer: “no”). The classification of the results distinguished between “insufficiently informed” (zero or one correct answer), “moderately well informed” (two or three correct answers) and “well informed” (four correct answers). Percentages were always calculated relative to the number of patients who responded to each statement or answered each question.

IBM SPSS Statistics for Windows, version 20.0 (Armonk, NY: IBM Corporation) was used for the statistical analysis. The data were analyzed in relation to general frequency distributions. In view of the size of the group of patients, a chi-squared distribution of the test variables was assumed in order to check independence. Evidence of independence was assessed using a chi-squared test. The α significance level was set at 0.05.

## Results

Between January 2011 and January 2012, 2546 patients completed the questionnaire (Table 1). Among 2363 patients for whom information about their presenting condition was available, 211 (8.9 %) had presented due to a gynecological cancer; 550 (23.3 %) due to breast cancer; 832 (35.2 %) due to pregnancy or birth; 82 (3.5 %) for fertility treatment; 198 (8.4 %) due to endometriosis; and 490 (20.7 %) due to a benign gynecological disease. The patients’ mean age was 42.1 years (range 14–96 years).

**Table 1:** Characteristics of the patients

Characteristics		n	%
Overall group		2546	100.0
Age	Mean 42.1 y (range 14–96 y)		
	Information	1758	100.0
	<21 y	29	1.6
	21–30 y	332	18.9
	31–40 y	601	34.2
	41–50 y	350	19.9
	51–60 y	229	13.0
	61–70 y	142	8.1
	71–80 y	63	3.6
	81–90 y	10	0.6
	>90 y	2	0.1
	No information	788	–
Presenting condition	Information	2363	100.0
	Pregnancy/birth	832	35.2
	Gynecological cancer	211	8.9
	Breast disease	550	23.3
	Fertility treatment	82	3.5
	Endometriosis	198	8.4
	Benign gynecological disease	490	20.7
	No information	183	–
Educational level	Information	1929	100.0
	No graduation	23	1.2
	High school	307	15.9
	Secondary school	694	36.0
	Higher education	300	15.6
	University	510	26.4
	Doctoral degree	90	4.7
	Postdoctoral degree	5	0.3
	No information	617	–
Postdiagnosis period	Mean 2.0 y (range 1–7 y)		
	Information	689	100.0
	0–1 y	351	50.9
	1–2 y	209	30.3
	2–3 y	34	4.9
	3–4 y	25	3.6
	4–5 y	17	2.5
	5–6 y	51	7.4
	6–7 y	2	0.3
	No information	1857	–

**Fig. 1:**
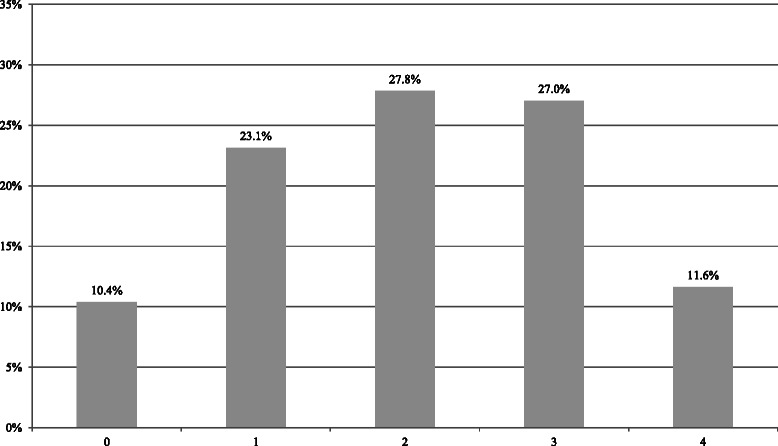
Analysis of patients’ knowledge about clinical studies (relative frequencies of numbers of correct answers)

**Fig. 2:**
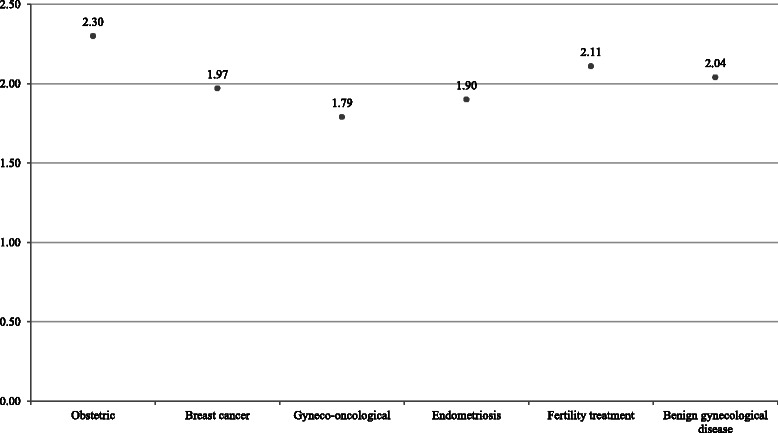
Means for the sum of correct answers from each patient relative to the patients’ presenting conditions

With regard to the patients’ level of knowledge about clinical studies, the responses to the nine questions in the first section of the questionnaire are presented in Table 2. The individual questions were answered by the patients with different frequencies (range *n* = 1914–1971). Statistical analyses did not identify any subgroups that had answered all of the questions or omitted to answer all of the questions in any section. The four clearly true or false statements were analyzed using a score (the sum of correct answers given by each patient) as an indicator of existing knowledge about clinical studies (Fig. 1). The score was calculated from a total of 2005 patients. On average, the patients assessed 2.06 statements correctly; 233 patients (11.6 %) had all four answers right, 543 (27.0 %) had three answers correct, 558 (27.8 %) had two answers right, 464 (23.1 %) had one answer right, and 208 (10.4 %) had no correct answers. Patients who presented in the department due to pregnancy or birth had an average of 2.3 answers right; patients with gynecological diseases had 1.79; patients with breast cancer had 1.97; patients attending for fertility treatment had 2.11; patients with endometriosis had 1.9; and patients with benign gynecological diseases had 2.04 (*P* < 0.001) (Fig. 2). The level of education showed a significant effect on the results in the knowledge scale (*P* < 0.001). A higher educational level was associated with more correct answers (Table 3). With regard to the analysis of age, the overall group was divided into nine age groups. The results for the key questions showed significant differences relative to age (*P* < 0.001). Participants aged 31–40 years responded to most questions correctly. The mean for correct statements decreased with increasing age (Table 4).

**Table 2:** Summary of statements to assess patients’ level of knowledge about the design and conduct of studies (questions on the rating scale are marked in bold type)

Statement	Yes % (n)	Don’t know % (n)	No % (n)	Total
Research studies are used when traditional medicine has failed.	18.5 (365)	28.2 (555)	53.3 (1051)	1971
Taking part in research studies involves greater risks for patients than standard therapy.	17.1 (336)	85.9 (685)	48.0 (943)	1964
The quality of treatment in research studies is much better.	21.9 (429)	54.9 (1074)	23.2 (453)	1956
In a clinical study, the physician treating you will ensure that you receive the treatment that is the best possible for you.	47.8 (914)	37.9 (726)	14.3 (274)	1914
**Do you know what the word “randomization” means?**	20.1 (396)	–	79.9 (1573)	1969
**Clinical studies make it possible to find out whether the standard treatment or a new method is more effective**.	83.7 (1640)	14.7 (288)	1.6 (32)	1960
**Research studies cannot be used in the treatment of severe diseases**.	8.4 (164)	43.1 (839)	48.5 (945)	1948
Clinical studies test the effects of procedures, drugs, or treatments that no one is yet certain about.	51.5 (1004)	23.9 (466)	24.6 (480)	1950
**After you agree to take part in a study, withdrawing from it later on is no longer possible**.	4.4 (85)	35.9 (699)	59.7 (1161)	1945

**Table 3:** Results from the knowledge rating scale relative to educational level

Highest educational level	n	Mean	Standard deviation	Median	*P*
No qualifications	23	1.48	1.201	1	<0.001
High-school certificate	299	1.34	1.011	1	
Secondary school	671	1.87	1.044	2	
Higher education	294	2.13	1.063	2	
University	498	2.57	1.081	3	
Doctoral degree	88	3.38	1.043	4	
Postdoctoral degree	5	3.20	1.304	4	
*Total*	1878	2.11	1.170	2

**Table 4:** Results from the knowledge rating scale relative to age

Age group	n	Mean	Standard deviation	Median	*P*
<21 years	21	1.43	1.287	1	<0.001
21-30 years	268	2.05	1.107	2	
31–40 years	520	2.36	1.133	2	
41–50 years	283	2.11	1.203	2	
51–60 years	183	1.98	1.160	2	
61–70 years	110	1.85	1.060	2	
71–80 years	52	1.33	0.985	1	
81–90 years	7	1.00	1.000	1	
>90 years	1	0.00	–	0	
*Total*	1445	2.11	1.164	2	

In the overall group (*n* = 1985), 91.6 % of the patients regarded research studies as useful (Table 5). However, only 52.8 % of the patients (*n* = 1992) stated that they would consider taking part in studies themselves (Table 5). Research studies were regarded as more useful with increasing educational level: 82.6 % (*n* = 19) without educational qualifications and 85.0 % (*n* = 250) with a high-school certificate, versus 96.5 % (*n* = 475) with a university degree (*P* < 0.001). This significant influence was also observed in relation to the question of whether patients would consider taking part in studies themselves: 26.1 % (*n* = 6) without qualifications and 43.8 % (*n* = 130) with a high-school certificate, versus 59.9 % (*n* = 299) with a university degree (*P* < 0.001). In the different age groups, participants aged 31–40 years included the highest proportion who regarded clinical studies as useful: 95.2 % (*n* = 491) thought studies were beneficial. Very young and older patients had a less positive view: 71.4 % (*n* = 15) of patients < 21 years, 88.7 % (*n* = 47) of patients aged 71–80 years, and 71.4 % (*n* = 5) of patients aged 81–90 years (*P* = 0.001). These views were also reflected in the patients’ actual willingness to participate in a trial: 59.3 % (*n* = 169) of those aged 41–50 years and 61.6 % (*n* = 109) of those aged 51–60 years were willing to participate, while only 44.6 % (*n* = 120) of those aged 21–30 years and 42.9 % (*n* = 3) of women aged 81–90 years would consider participation (*P* = 0.001).

**Table 5:** Summary of opinions in the group regarding the value of clinical studies and factors potentially influencing participation

Question	Yes % (n)	Don’t know % (n)	No % (n)	Total
Do you think research studies are useful?	91.6 (1819)	7.8 (155)	0.6 (11)	1985
Would you be willing to take part in research studies?	52.8 (1052)	30.5 (608)	16.7 (332)	1992
Would a recommendation to take part in research studies by public institutions such as German Cancer Aid or the German Cancer Society influence your willingness to take part?	33.4 (634)	32.7 (622)	33.9 (645)	1901
Would financial compensation influence your willingness to take part?	11.6 (223)	24.1 (462)	64.3 (1233)	1918
Do you think that declining to take part in research studies could have negative effects on the treatment you receive?	2.6 (50)	23.8 (458)	73.6 (1418)	1926
Do you think that declining to take part in research studies could have negative effects on your relationship with the physician treating you?	2.0 (38)	22.9 (441)	75.1 (1447)	1926
Would you like to receive more information about clinical studies that are available?	29.1 (548)	17.5 (330)	53.3 (1003)	1881
Is the availability of research studies at our hospital one reason why you came here for treatment?	7.8 (149)	5.1 (98)	87.1 (1673)	1920

Among patients who were willing to participate, 15.9 % would be willing to take part in drug tests, 75.2 % in surveys, 54.7 % in tests of new treatment methods, and 32.5 % in studies on ways of improving diagnosis (*n* = 1351). Patients with breast cancer (*n* = 271) were most likely to take part in a study (64.4 %), followed by 55.0 % of patients with endometriosis (*n* = 160), 52.7 % of those with a gynecological cancer (*n* = 165), 49.6 % of those with a benign gynecological disease (*n* = 379), 48.6 % of those attending for pregnancy or birth (*n* = 716), and 48.4 % of those receiving fertility treatment (*n* = 64) (*P* = 0.001).

Only 11.6 % of the patients (*n* = 1918) would be influenced in favor of taking part in research studies if they were to receive financial remuneration for it (Table 5). Patients receiving fertility treatment were most likely, at 21.9 %, to be positively influenced by financial remuneration (*n* = 64), while patients with breast disease (*n* = 412) and gynecological cancers (*n* = 154) were least likely, at 4.1 % and 8.4 %, respectively (*P* = 0.001). Educational level did not have a significant effect on willingness to participate relative to financial remuneration (*P* = 0.265). Financial remuneration showed a linear correlation with age: 17 % (*n* = 44) of women aged 21–30 years and 14.0 % (*n* = 71) of those aged 31–40 years stated that it would have a positive influence on their participation. This proportion decreased with increasing age, down to 2.1 % of patients aged 71–80 years and none of those who were older (*P* < 0.001).

A total of 494 of 1919 patients (25.7 %) would prefer to make any decisions about study participation alone; 1121 (58.4 %) would prefer to make the decision themselves after careful consultation with the physician treating them, while 279 patients (14.5 %) would make the decision jointly with their physician. Twenty patients (1.0 %) would allow the physician to make the decision about study participation after the patient had been asked for her own opinion. Five patients (0.3 %) wanted the physician alone to make the decision.

The preferred method of obtaining information about a study was through a specialist physician, in 81.3 % of the patients, followed by receiving information from the family physician in 5.9 % (*n* = 1383). Among patients with high-school certificates, 75.5 % (*n* = 142) wished to obtain information about a study from a specialist and 8 % (*n* = 15) from the family physician. In those with university degrees, 84.6 % (*n* = 55) preferred specialists and only 3.1 % (*n* = 2) preferred their family doctor (*P* = 0.021). Age did not have a significant influence (*P* = 0.191).

When receiving information about the nature of a study in which they might participate, 1763 of 1838 patients (95.9 %) wanted to receive all available information about the study, while only 63 (3.4 %) in principle wanted only as little information about the study as possible. The wish to receive the maximum information about a study increased significantly with a higher level of education — high-school certificate 91.8 % (*n* = 247) versus university degree 97.9 % (*n* = 466; *P* = 0.006) — and decreased continuously with increasing age: 97.2 % (*n* = 240) of patients aged 21–30 years versus 87.0 % (*n* = 40) of those aged 71–80 years (*P* < 0.001).

A total of 1283 patients (37.1 %) thought that future generations would benefit most from their participation in the study; 929 (26.9 %) thought that they themselves would benefit most as patients; 459 (13.3 %) thought the physician would benefit most; 431 (12.5 %) thought the pharmaceutical industry would benefit most; 259 (7.5 %) thought hospitals would benefit most; and 94 (2.7 %) thought that the health insurance company would benefit most (multiple answers were possible).

Only 2.6 % of all patients (*n* = 1926) feared that declining to participate in a study would have negative effects on the way in which they were treated, and only 2.0 % of all patients (*n* = 1926) feared negative effects on their relationship with the physician treating them (Table 5). Educational level did not have a significant influence on the patients’ view that a negative relationship with the treating physician might develop if they declined study participation (*P* = 0.762); 4.8 % (*n* = 14) of patients with high-school certificates were afraid that there might be a negative effect on treatment, while only 1.4 % (*n* = 7) of the patients with university degrees feared this (*P* < 0.001). Anxiety regarding a negative influence on their treatment if they declined to participate was expressed more frequently by younger patients: 2.0 % (*n* = 10) of those aged 31–40 years versus 0 % of all patients > 70 years (*P* = 0.001). Similar results were observed for the patients’ relationship with the physician (2.4 % versus 0 %; *P* = 0.002).

In all, 29.1 % of the patients (*n* = 1881) wanted to receive more information about available clinical studies (Table 5). More information about available studies was wanted particularly (46.2 %) by patients receiving fertility treatment (*n* = 65), followed by 37.2 % of patients with breast cancer (*n* = 409), 36.1 % of patients with endometriosis (*n* = 147), and 30.8 % of patients with a gynecological cancer (*P* = 0.001). Educational level did not have a significant effect on patients’ desire to receive further information about available clinical trials (*P* = 0.189). Patients aged 51–60 years had the strongest desire to receive further information about available studies (41.1 %, *n* = 72). This decreased continuously in lower and higher age groups: 24.3 % (*n* = 63) of patients aged 21–30 years and 31.1 % (*n* = 14) of women aged 71–80 (*P* = 0.011).

Only 7.8 % of the patients (*n* = 1920) presented at the Department of Gynecology at Erlangen University Hospital specifically because of the availability of research studies (Table 5).

## Discussion

It has been shown in a wide variety of studies that treatment in the framework of research studies has advantages for patients — partly because more intensive clinical care and monitoring is provided, and also because there are positive psychological effects involving better coping with the disease and the ability to receive the latest innovative therapies [3]. In addition, research studies lead to increased knowledge about the pathogenesis and risk factors in diseases [6]. In a questionnaire study conducted among American oncologists by Somkin et al. in 2005, 67 % of the physicians stated that the treatment of patients in clinical studies was always the treatment of choice [7]. Despite this, recruitment rates of research studies are well below 10 % in many certified centers [8]. The study recruitment rate for all certified German breast cancer centers in 2012 showed a median of 11.3 % [9]. It is therefore clearly necessary to optimize recruitment strategies. These strategies should be based on the patients’ attitudes and knowledge about clinical studies.

The present study shows that patients are on average “moderately well-informed” in relation to clinical studies, according to a score based on four questions used as a separate indicator of existing knowledge about clinical studies — i.e., the patients answered an average of 2.06 questions correctly. Middle-aged patients, those with a high educational level, and those presenting with pregnancy or endometriosis had the best levels of information about studies.

A report from Australia calculated a knowledge score on the topic of clinical studies among 50 outpatient internal medicine/oncology patients [5]. The findings showed that 51.0 % of the patients agreed with the statement that a clinical study discovers whether one method works better than another. A systematic review and meta-analysis included 103 studies analyzing patients’ knowledge about different aspects of clinical trials. The pooled percentage of participants was 62.9 % for knowing that treatments were being compared [10]. In comparison, 83.7 % of the patients in the present study agreed that this statement was true. By contrast, 74.0 % of the Australian group were certain that the physician treating them would ensure that a patient taking part in a study would receive the best possible treatment — whereas in the present study only 47.8 % agreed with this. There was a correspondence in relation to the statement that clinical studies are only used when the situation is hopeless (18.0 % versus 18.5 %). The view that clinical studies test procedures etc. that no one is yet certain about was agreed to by only 19.0 % in the Australian group, in comparison with 51.5 % of the patients in the present study. Evidently, patients in the present group were much more uncertain in relation to the individual questions listed. Both questionnaires used the knowledge rating scale tool. Studies have identified a positive connection between patients’ willingness to participate and the number of correct answers regarding knowledge about clinical studies [8, 11]. By comparison, the median score in the present group was two (out of four key questions), with the average lying at 51.5 %, while in Australia it was three out of seven questions. The meta-analysis showed that the proportion of participants who understood different components of clinical trials varied from 52.1 % to 75.8 % [10].

Bergenmar and colleagues have factors associated with patients’ knowledge and perceived understanding of clinical trials by using a knowledge score test in 268 patients who consented to participate in a clinical trial [12]. No significant associations were found between knowledge and clinical and socio-economic factors. In contrast to these findings, the present study shows significant influences relative to age, presenting condition, and educational level; this may be explained by the larger number of participants and a better ability to detect statistical differences. The positive influence of lower age and higher education on knowledge has been confirmed by other studies [11].

Research studies were considered useful by 91.6 % of the patients, but only 52.8 % were actually willing to take part in studies themselves. The discrepancy between general approval of clinical studies and patients’ actual or real willingness to take part in them has also been reported in other studies [8, 13, 14]. Ellis et al. reported that women who were willing to consider taking part in a randomized trial were younger and had a higher educational level [8]. Both of these aspects were confirmed by the present study, but there was also a group of very young women who showed less willingness to participate.

A patient’s decision on whether to take part in a study is influenced more by the potential disadvantages than by the potential advantages [14]. The majority of the respondents have a negative image of clinical studies and of the potential disadvantages [14, 15]. Frequently mentioned reasons for not taking part in studies include, for example, a fear of being used as a guinea-pig [8], the increased personal effort involved [8, 16], and increased personal risk [16]. Positive reasons for taking part in studies that are mentioned often include making a contribution to scientific progress [13, 17], the hope that one will receive what is currently the best form of treatment [8] or better treatment and care in comparison with the standard therapy [18], and benefits for future generations [8, 13]. The present study shows that a total of 37.1 % of the patients considered that future generations would be likely to benefit most from their participation in a study; by contrast, only 26.9 % thought that they might benefit most as patients themselves.With regard to external influencing factors, it has been shown that recommendations by independent information services often have a positive effect on willingness to participate in studies [5, 8]. This effect was only moderate in the present group, with only one in three patients being positively influenced by this.

Financial incentives only influenced around one in 10 patients in this group in their decision for or against taking part in studies. Reasons for the particularly positive reaction to a financial bonus among women receiving fertility treatment (21.9 %) are likely to be the high costs of this form of treatment and the high level of the excess charge for it that is not met by health insurance companies. Younger patients were also more often influenced by financial remuneration. This may be explained by the general financial burdens faced by younger people (e.g., relative to starting out on their careers, obtaining mortgages for houses, and caring for children).

The great majority of the present group of patients wanted to make the decision about whether or not to participate in a study after consultation with the physician treating them — but nevertheless independently. This finding is also reflected in other studies [5, 15]. Women who have an active decision-making style are more likely to take part in studies than women with a passive decision-making style [8]. Satisfaction with decision-making and subjective informed consent is strongly associated with fewer regrets about decisions [19].

The amount of information desired by patients is also important in the context of autonomy when participating in a study. The majority of patients in the present group (95.9 %) wanted to receive all of the available information during the consultation process. A similar picture has also been described in other studies [5, 15]. Patients who do not believe that they have fully understood the implications of a study may ultimately feel regret about their decision to participate [19].

Altruistic motivations generally play an important role in study participation, as has often been shown in other studies [20, 21]. In addition, the idea that there may be benefits for future patients is associated with high levels of knowledge [22]. Verheggen et al. found that altruistic motives weakened the expected risks of study participation and led to the extra time involved being seen as less burdensome, which in turn led to higher participation rates [16]. In the present group, benefits for the participating patient were regarded as being only secondary; this may be due to the supposed risks and disadvantages of potential participation in a study. This attitude has also been observed in other studies [14]. Although having an altruistic attitude is positively associated with an individual’s potential participation in research studies, it is not a reliable predictive factor [8]. It is only when a benefit for study participants themselves is seen that study participation becomes probable [13, 23].

These findings — both from the current study and also from the numerous other studies cited here — offer some points of departure for optimizing study planning, study design, patient recruitment, and also the conduct of research studies and processing of the results. To begin with, the way in which information is communicated to patients should be optimized — e.g., through early provision of information brochures, posters, etc., in order to dispel incorrect ideas and thus reduce or prevent anxieties [5, 10]. Presentations using audiovisual media can have positive effects on the quality of information and may increase willingness to participate, but evidence on this is still weak and further research is necessary [24].

In addition, the personal relationship of trust between the physician and the study participant, as well as the availability of personal discussions, should be emphasized [25]. The specialist physician treating the patient was the preferred contact person in the present group (81.3 %) for providing information about a study. A sensitive informed consent discussion strengthens the physician–patient relationship, and a positive relationship is regarded as a predictive factor for participation in research studies [26]. Training programs for physicians to help develop special communication skills can lead to more positive attitudes to clinical trials on the part of patients [27].

Involvement of patients’ family physicians would also be an important way of contacting patients in connection with participation in research studies. It is also important to estimate the financial and time pressure involved for study participants and to provide support when appropriate. This might take the form of financial grants for travel costs or treatment costs, for example [28].

The present study has limitations connected with the composition of the study group, with a wide variety of subgroups. There was also a wide range of responses for each question with regard to the assessment of the patients’ level of knowledge about the design and conduct of studies. One reason for this may have been the comprehensibility of some of the questions. There were no defined subgroups that failed to respond to all of the questions. A further limitation of the present study is the reliability and validity of the instruments used. Although the knowledge scale has been used in earlier studies [5] and differences between results can be discussed on a reliable basis, the questionnaire itself was designed for this study and validation is still lacking. On the other hand, the comprehensibility of the questionnaire was checked using interviews with the first 20 participants. Despite this, the overall group was very large, compensating at least in part for the limitations mentioned. Another limitation is the fact that this study only included female participants; however, other studies have not identified any differences, or only minor differences, between male and female participants [12, 22, 29].

## Conclusions

This study shows that women with gynecological diseases have only moderate knowledge about clinical studies. In addition, their knowledge and willingness to participate in research studies are strongly dependent on age (*P* < 0.001), educational level (*P* < 0.001), and presenting condition (*P* < 0.001). Middle-aged patients, those with a high educational level, and those who presented with pregnancy or endometriosis, were informed best about research studies. A majority of the patients considered that clinical studies were useful, but only a few patients were also willing to take part in them. Various factors may influence patients’ willingness to participate in medical studies and their attitudes toward the design and conduct of such studies. General public information should be provided about the background to research studies. To increase the rates of participation in clinical studies, the patients need more intensive information about the benefits to themselves that the relevant study can offer. Physicians need to respond in a targeted way to patients’ questions and anxieties. Future research should concentrate on the development of tools (e.g., web-based tools) to optimize individualized information provision about the background of research studies in ways that are based on age, educational level, and health condition. Tools of this sort and the effect of approval for them from medical professional associations should be evaluated in the framework of future clinical trials.
